# Feasibility of Food FARMacia: Mobile Food Pantry to Reduce Household Food Insecurity in Pediatric Primary Care

**DOI:** 10.3390/nu14051059

**Published:** 2022-03-03

**Authors:** Jennifer A. Woo Baidal, Dodi Meyer, Ivette Partida, Ngoc Duong, Alyson Rosenthal, Emma Hulse, Andres Nieto, on behalf of the HERALD Collaborative

**Affiliations:** 1 Division of Pediatric Gastroenterology, Hepatology, and Nutrition, Department of Pediatrics, Columbia University Vagelos College of Physicians & Surgeons, New York, NY 10032, USA; ip2359@cumc.columbia.edu (I.P.); nqd2000@cumc.columbia.edu (N.D.); 2 NewYork-Presbyterian Morgan Stanley Children’s Hospital, New York, NY 10032, USA; ddm11@cumc.columbia.edu; 3 Division of Child and Adolescent Health, Department of Pediatrics, Columbia University Vagelos College of Physicians & Surgeons, New York, NY 10032, USA; 4 West Side Campaign Against Hunger, New York, NY 10032, USA; arosenthal@wscah.org; 5 Division of Community and Population Health, NewYork-Presbyterian, New York, NY 10032, USA; emh9022@nyp.org (E.H.); nietoan@nyp.org (A.N.)

**Keywords:** Food FARMacia, food insecurity, intervention, food pantry, pediatric primary care, obesity, feasibility

## Abstract

Despite recommendations for systematic food insecurity screening in pediatric primary care, feasible interventions in clinical settings are lacking. The goal of this study was to examine reach, feasibility, and retention in Food FARMacia, a pilot clinically based food insecurity intervention among children aged <6 years. We examined electronic health record data to assess reach and performed a prospective, longitudinal study of families in Food FARMacia (May 2019 to January 2020) to examine attendance and retention. We used descriptive statistics and bivariate analyses to assess outcomes. Among 650 pediatric patients, 172 reported household food insecurity and 50 registered for Food FARMacia (child mean age 22 ± 18 months; 88% Hispanic/Latino). Demographic characteristics of Food FARMacia participants were similar to those of the target group. Median attendance rate was 75% (10 sessions) and retention in both the study and program was 68%. Older child age (retention: age 26.7 ± 18.7 months vs. attrition: age 12.1 ± 13.8 months, *p* = 0.01), Hispanic/Latino ethnicity (retention: 97% vs. attrition: 69%, *p* < 0.01), and larger household size (retention: 4.5 ± 1.1 vs. attrition: 3.7 ± 1.4, *p* = 0.04) correlated with retention. A clinically based mobile food pantry pilot program and study reached the target population and were feasible.

## 1. Introduction

Household food insecurity and other social determinants of health (SDoH) are increasingly recognized as playing key upstream roles in etiologies and prevention of obesity and other chronic diseases, particularly in disproportionately burdened populations [1]. The National Academy of Medicine [2], American College of Physicians [3], American Academy of Pediatrics [4], and others have recommended incorporating screening for household food insecurity into primary care. Despite calls to integrate routine screening of household food insecurity in clinical practice, feasible and effective clinical interventions to address household food insecurity for population health management are limited [5].

Recent evidence shows that households with children have less income for food and are more likely to use emergency food services than those without children since the onset of COVID-19 [6]. Additionally, racial/ethnic disparities in access to emergency food services are widening [6]. With persistent unemployment, intermittent school closures, and increasing food insecurity prevalence subsequent to the onset of the COVID-19 pandemic, an urgent need to provide emergency food assistance to children disproportionately burdened by household food insecurity during critical periods in life exists. In the first years of life, children are particularly vulnerable to stressors [7,8,9,10,11]. They also attend frequent routine health care visits, making primary care a promising setting to intervene to reduce household food insecurity. The American Academy of Pediatrics (AAP) and the Food Research & Action Center (FRAC) Toolkit for Pediatricians outlines potential clinical interventions that pediatricians can employ in clinical settings, but evidence for the effectiveness of timely clinical interventions is needed to determine which interventions should be disseminated [12].

In the United States (U.S.), federal nutrition programs attenuate food insecurity and promote healthy nutrition [13]. Referrals to nutrition programs are a mainstay of pediatric care [12]. However, enrollment into programs is not immediate, eligibility requirements must be met, and benefits may be insufficient. Most prior or ongoing research on clinically based food insecurity interventions have focused on brick-and-mortar food pantries, produce prescription programs, or medically tailored nutrition interventions among elderly adults, uninsured adults, or adults with specific chronic diseases such as cancer or type 2 diabetes [14,15,16,17,18]. In order to develop and test effectiveness of food security interventions in clinical settings, we first must identify feasible interventions.

Food pantries may be feasible clinically based interventions. Evidence supports that food pantries may reduce food insecurity, and pantry-based interventions that increase access to healthy food can improve dietary quality [19,20,21,22]. Cost, space, and staff constraints can preclude implementation of traditional food pantries in areas with high food insecurity prevalence [19,20,21,22]. Mobile food pantries—programs that use vans or trucks to bring food into communities—are emerging as one potential solution for increasing access to food [23]. Despite their increasing availability, little research examines mobile food pantry implementation and feasibility in clinical settings to reduce household food insecurity among patients. 

The overall goal of this study is to examine the feasibility of Food FARMacia, a novel pilot program using a choice-based mobile food pantry among families with a child aged <6 years receiving routine healthcare in a pediatric primary care setting. Specifically, we aimed to examine the extent to which the pilot program would reach the target population. We additionally sought to describe the feasibility of recruitment, enrollment, and retention in the Food FARMacia and a longitudinal research study for 6 months. Among those enrolled into the research study, we also aimed to explore the patient and household characteristics correlated with retention and attrition in the program at 6-month follow-up.

## 2. Materials and Methods

### 2.1. Study Design, Setting, and Participants

In this observational study, we used electronic health record (EHR) data to examine program reach, program data to report intervention fidelity and dose received, and longitudinal study data to report on feasibility of recruitment and data collection over 6 months. For the overall study sample, we included all patients aged <6 years with a routine pediatric primary care visit at Washington Heights Family Health Center (WHFHC), NewYork-Presbyterian (NYP). NYP is an academic health care system affiliated with Columbia University Vagelos College of Physicians and Surgeons and Weill Cornell Medicine. WHFHC is part of the NYP Ambulatory Care Network and is part of the Columbia University Irving Medical Center campus located in Washington Heights in Northern Manhattan. Compared to NYC as a whole, Northern Manhattan has 2.5-fold higher proportion of Hispanic/Latino residents, with 73% Hispanic or Latino, 11% Black or African American, and 11% white [24]. Washington Heights has a median household income of USD 34,777, which is lower than the New York City median household income of USD 52,259 [24]. Patients with a well-child visit at WHFHC between 2 April 2019–2 August 2019 and at least one screening questionnaire for household food insecurity completed within this timeframe were eligible for inclusion. 

Household food insecurity screening is routinely completed at well-child visits as part of ANCHOR (Addressing the Needs of the Community through Holistic, Organizational Relationships), the Accountable Health Communities (AHC) model at NYP/CUIMC. The AHC health-related needs screening tool includes the 2-item Hunger Vital Signs™, a validated food insecurity screening tool [25,26]. We defined household food insecurity as a parental response of “often true” or “sometimes true” on either question [26]. We defined the Food FARMacia target population as patients aged <6 years with household food insecurity identified with the screening tool.

Patients were eligible for registration in the Food FARMacia if they: (1) had household food insecurity documented in the electronic health record or reported during their clinical encounter; (2) were a patient aged <6 years; and (3) had a parent/guardian who agreed to attend a food assistance program including free-of-charge food selection twice monthly. Participants were unenrolled from the program if they stated a desire to leave the program or if they did not attend two consecutive food selection sessions. Additional participants were registered to fill vacated program spots. 

Food FARMacia participants were eligible for enrollment in the longitudinal survey study if parents or legal caretakers were at least age 18 years and could respond to questions in English or Spanish. Program staff referred potentially eligible participants who wished to enroll in the longitudinal study to on-site study staff. Study staff obtained written informed consent. Study staff administered surveys at baseline and 6-month follow-up in English or Spanish in-person or by telephone. All procedures were approved by the CUIMC Institutional Review Board.

### 2.2. Food FARMacia Pilot Program: Mobile Food Pantry Intervention to Reduce Food Insecurity

The Food FARMacia pilot program took place at WHFHC, a clinical practice site of the NewYork-Presbyterian Ambulatory Care Network, from 28 May 2019–7 January 2020. Choosing Healthy and Active Lifestyles for Kids™ (CHALK), a NYP community-based obesity prevention program in partnership with Community Pediatrics at CUIMC, implemented the pilot clinical program as part of routine pediatric primary care. NYP CHALK staff registered families eligible for the program. 

**Figure 1:**
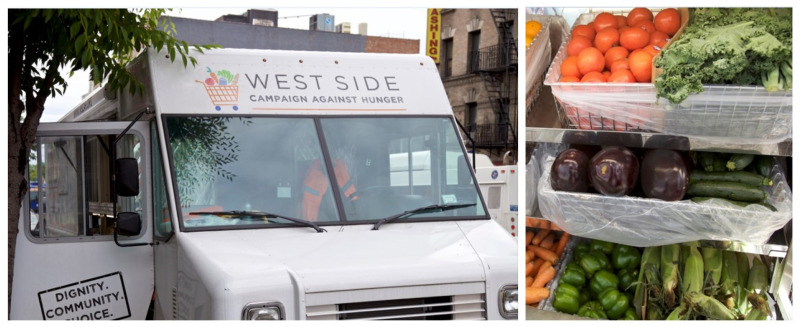
Food FARMacia: Front and side view of mobile food pantry truck.

The Food FARMacia was operated by West Side Campaign Against Hunger (WSCAH), a NYC-based emergency food provider with over four decades of experience providing healthy food and supportive services. In the Food FARMacia, we used a mobile food pantry based upon WSCAH’s customer-choice model that follows USDA MyPlate guidelines. On a twice-monthly basis, the Food FARMacia was administered at the WHFHC. At each contact, families selected food from the Food FARMacia mobile food truck without charge (Figure 1). The food amounted to approximately 12 meals per household member for up to 5 household members. Program registrants received reminders of food selection sessions prior to each session by phone call or text message. In addition to food selection, program components included: (1) cooking demonstrations led by a nutritionist at food selection sessions focused on preparation of healthy and seasonal meals; (2) referrals and assistance with enrollment in supplemental nutrition programs; and (3) provision of round-trip public transportation fare to attend food selection sessions. The program registered participants to a target of serving 150 individuals per session because of food truck storage capacity.

### 2.3. Outcome Measurements

#### 2.3.1. Reach and Feasibility of Food FARMacia Pilot Program and Longitudinal Study

To assess program reach, we extracted EHR data for all clinical encounters at WHFHC during the program registration period for the overall patient population aged <6 years. We examined individual and household demographic characteristics for patients aged <6 years, those with household food insecurity (target population), and those registered for the Food FARMacia pilot program. We examined child age, child sex, child race/ethnicity, household size, and annual household income to assess the extent to which the program reached the target population. We also examined self-reported high utilization of emergency department (ED) visits (2 or more ED visits in past 12 months) and health-related social needs (HRSN) according to type and number using the AHC tool [25].

#### 2.3.2. Feasibility of Food FARMacia Pilot Program and Longitudinal Study

For program feasibility, we assessed program fidelity and participant dose received using data collected by Food FARMacia program staff for the subset of participants enrolled in the longitudinal study. We defined program fidelity as the number of sessions (doses) planned and delivered by the program. We defined participant dose received as program attendance rates over 6 months using the proportion of sessions attended per sessions delivered. Additional attendees were registered periodically to maintain maximum attendance at each food selection which could provide food for about 50 families (total 150 individuals). Thus, not all participants had the same number of doses possible.

To assess the characteristics of Food FARMacia participants according to retention or attrition at 6-month follow-up, we used baseline responses to the survey questions administered by study staff about parental respondent sex, language use, highest educational level, and enrollment in WIC or SNAP. We quantified survey completion rates at 6 months after enrollment. 

### 2.4. Statistical Analysis

We reported frequencies (%) of process and feasibility measures. To assess the extent to which the pilot program reached the target population, we also examined frequencies (%) and means (SD) of demographic characteristics and HRSN among the overall patient population with food insecurity, those registered in Food FARMacia, and those enrolled in the longitudinal observational study. We used descriptive statistics to report Food FARMacia attendance rates among those enrolled in both the program and the longitudinal study.

Among those in the Food FARMacia longitudinal observational study, we used the Wilcoxon rank-sum test (for continuous variables) and Fisher’s exact test (for categorical variables) as appropriate to explore differences in baseline characteristics between those who remained in the Food FARMacia program at 6 months and those with program attrition. We evaluated each characteristic in a separate hypothesis test and considered *p* < 0.05 as statistically significant in these exploratory analyses. All analyses were performed in R Statistical Software (version 4.1.1). 

## 3. Results

### 3.1. Reach of Food FARMacia Pilot Program

**Figure 2:**
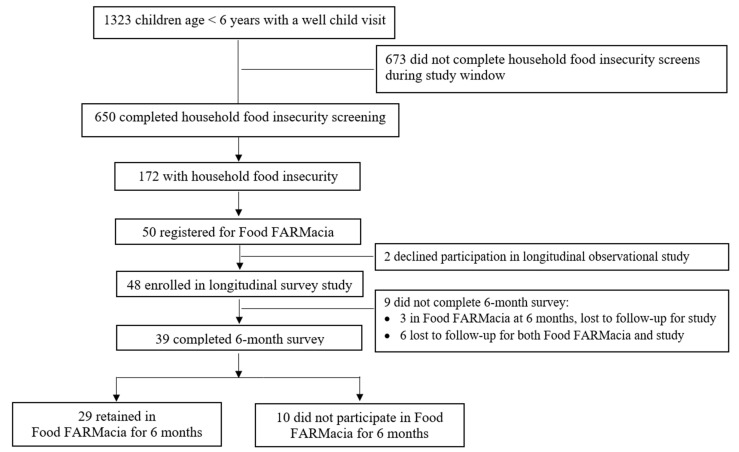
Flowchart of study flow for Food FARMacia pilot program and research study retention. Data from a single clinic in a multi-site ambulatory clinical practice in Washington Heights, New York City (2 April 2019–2 August 2019).

Among 650 patients with a child aged under 6 years and a pediatric primary care visit at WHFHC in the study period, 172 (26.5%) had household food insecurity and comprise the target population for Food FARMacia (Figure 2). Of those with household food insecurity, 50 (29%) registered in the Food FARMacia pilot program. Registration stopped when the program was at maximum capacity.

Table 1 shows baseline characteristics of participants according to the overall sample, the subset with household food insecurity, and the subset with household food insecurity who registered for Food FARMacia. Overall, Food FARMacia registrants had similar demographics as the target population. The majority of the target population and Food FARMacia registrants reported Hispanic/Latino ethnicity and annual household income less than USD 20,000. Average household size, ED utilization, household food insecurity, utility help needs, and safety concerns were similar between groups. Food FARMacia participants reported lower prevalence of kitchen problems (defined as either reporting non-functional stove or oven, or having pests such as bugs, ants, or mice) and transportation needs than the target population.

### 3.2. Feasibility of the Food FARMacia Pilot Program and Longitudinal Study

For program fidelity, 100% of the planned Food FARMacia pilot program sessions occurred during the pilot period. Because registration was ongoing to fill vacated program spots, six participants who registered after the initial registration period had fewer possible sessions to attend. Possible sessions for 6-months of participation ranged from 11 to 14 sessions. 

Among the 50 Food FARMacia pilot program participants (Figure 2), 48 (96%) enrolled in the longitudinal observational study. Of the 48 longitudinal study participants, 39 completed surveys at 6-month follow-up (81% retention). Because six of those who completed follow-up surveys were no longer in the Food FARMacia program, the 6-month retention rate was 68% for those who were retained in both the program and the survey study. 

**Figure 3:**
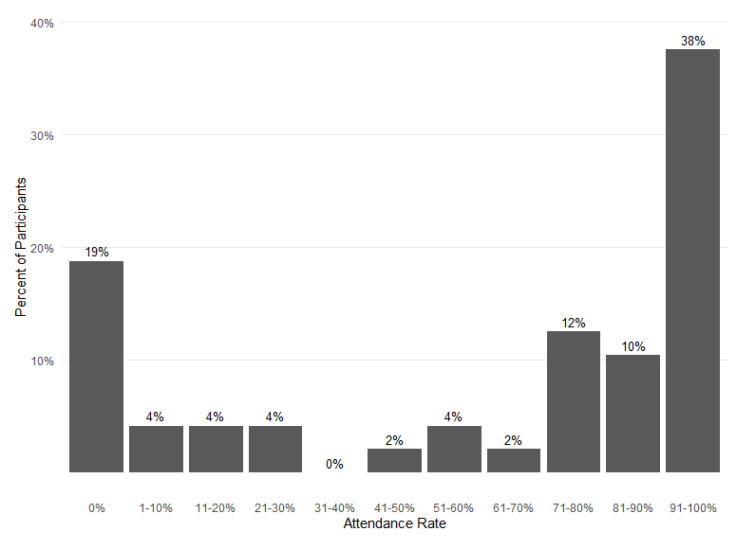
Food FARMacia pilot program 6-month attendance rates. Data from 48 families participating in the Food FARMacia program and longitudinal survey study.

Figure 3 shows attendance rates over 6 months of program participation for the 48 study participants. The mean attendance rate was 60% (eight sessions) and the median was 75% (ten sessions) over 6 months. Overall, most participants had attendance rates over 70%, but 19% (nine families) attended no sessions. Among those who attended at least one session, 74% attended more than 70% of sessions. 

Because some families attended no sessions after initial registration, registration was modified to include a provider referral. Of the nine families who attended no sessions, one registered through provider referral and eight registered in the initial registration period using direct registration by program staff without a referral. No other modifications occurred in the pilot program.

### 3.3. Characteristics of Participants Retained in The Food FARMacia Program

Table 2 displays baseline characteristics of Food FARMacia participants according to 6-month retention and attrition. In univariate analyses, those who remained in the Food FARMacia program had children with older age at baseline (26.7 months versus 12.1 months, *p* = 0.01), had larger household size (4.5 versus 3.7, *p* = 0.04), and more often reported Hispanic/Latino ethnicity (97% versus 69%, *p* < 0.01) compared to those with attrition at 6-month follow-up. Child sex, parental characteristics, household income, WIC enrollment, and SNAP enrollment were similar between groups. The type and number of health-related social needs also did not differ between groups (data not shown). 

## 4. Discussion

Among a cohort of 50 families with a child aged <6 years and household food insecurity, we found that the Food FARMacia pilot program reached the target population with high fidelity. The program was feasible and the attendance rate for most families was above 70%. We also found that recruitment and retention into a concomitant research study was feasible. Overall, our findings among predominantly Hispanic/Latino families support the feasibility of a clinically based mobile food pantry and research study among a population disproportionately burdened by household food insecurity and underrepresented in biomedical research studies. 

As more healthcare systems seek to address household food insecurity and other upstream determinants of health [2], research to inform feasible and effective clinically based interventions to reduce food insecurity is needed. In a systematic review and meta-analysis of food insecurity interventions in U.S. health care settings, among the 23 included studies, two directly provided food through an on-site food pantry [5]. In the first study, among 351 adult patients in a nested cohort study at cancer clinics in NYC, participants completed median three pantry visits over 4 months [14]. In a second cross-sectional study at a free clinic among adults, 43% received monthly boxes of food and 14% attended an offsite food pantry [27]. In our study, families attended a median of 10 food selection sessions and had 67% retention over 6 months, higher than other clinically based food pantry programs published to date. Some potential reasons for our higher attendance rate and retention may be related to the program registration, program design, use of reminders about food selection sessions, or the need of the patient population. A mixed-methods study of pediatric patients among a broader age group showed the feasibility of an in-clinic pantry [28]. In contrast to that study, we examined retention rates in a longitudinal program for families with a young child. 

Most prior or ongoing research on clinically based food pantries, fruit and vegetable prescriptions, or food delivery interventions have focused on elderly adults, uninsured adults, or adults with specific chronic diseases such as cancer or type 2 diabetes [14,15,16,17]. Our study adds to the literature by showing the need for and potential promise of implementing food security interventions among a population of pediatric patients who are attending routine well-child visits. Focusing on optimal growth and development starting early in life may ultimately reduce downstream chronic disease. 

Substantial evidence supports enrollment in WIC, SNAP, and other existing supplemental nutrition programs as effective means to reduce food insecurity, improve maternal–infant nutrition, and promote optimal infant growth [29]. In their meta-analysis, De Marchis and colleagues found that referrals to resources such as WIC and SNAP increase uptake of resource use [5]. Our pediatric primary care practices provide referrals to WIC and SNAP as standard of care when household food insecurity or other social needs are identified. In our cohort, all but one participant was enrolled in WIC at baseline. The persistence of household food insecurity despite WIC enrollment supports the need to go beyond referrals to WIC to provide timely emergency food assistance to curb household food insecurity in some urban families with young children. In our study, over half of participants were SNAP-enrolled at baseline. SNAP enrollment was similar among retention and attrition groups. Lack of baseline SNAP enrollment may be related to ineligibility, fear about use of public benefits among immigrants [30,31], or difficulties with completing necessary paperwork for maintaining enrollment. Although supplemental nutrition programs help reduce household food insecurity, our study sample experienced household food insecurity despite enrollment in benefits programs. The inadequacy of benefits, cost of high-quality nutrient-dense foods, and limited access to healthy foods in some communities may be reasons for these findings [32,33]. Recent increases in SNAP benefit allotments may help alleviate household food insecurity for some families, but strategies to remove eligibility restrictions and stigma are needed to enhance access to sustained benefits and help prevent food insecurity, adverse health outcomes, and forgone healthcare [34,35]. 

We also found that Food FARMacia participants had lower prevalence of transportation needs and kitchen problems than the target population. Patients with transportation needs may be unable to attend food selection sessions regularly and those with kitchen problems may be reluctant to participate in an emergency food program that requires food preparation. Alternatively, these findings could be a result of random variation in a pilot study with a small sample size. Better understanding of the multiple social needs of families with household food insecurity will help inform different intervention options.

We modified the program to allow for provider referrals. We found that a higher proportion of registered participants attended at least one food selection session after we implemented healthcare provider referrals compared to those directly registered by program staff. Our sample size is too small to draw conclusions about causality. However, healthcare providers are a trusted source of health information among parents of infants and young children, thus referrals provided by them may facilitate patient acceptance [36]. Reliance on healthcare provider referral alone could lead to referral bias. We used systematic screening of household food insecurity with systems-level practice changes to help reduce this potential bias.

We also found that families with older children, Hispanic or Latino ethnicity, and larger household size had lower rates of attrition versus counterparts in our exploratory analysis. In recent data from NYC, Hispanic/Latino families with children were among the groups most likely to suffer from economic challenges that resulted in food access limitations [6]. They also had less access to emergency food assistance [6]. Thus, the Food FARMacia model for household food insecurity intervention embedded in a pediatric primary care setting shows promise for reaching populations disproportionately burdened by household food insecurity to increase access to emergency food assistance.

## 5. Limitations

A limitation of this study is the observational nature in a small cohort of families from a single clinical site. Moreover, because we used electronic health record data for some data collection, missing data on some variables coupled with the small sample size limited the statistical power to identify differences in characteristics between those with retention or attrition at 6-month follow-up. We did not complete an evaluation or outcomes assessment in this study as the main goal of this study was to assess the feasibility of the pilot program and study. Despite these limitations, this pilot study achieved its overall goal of examining the feasibility of a mobile food pantry intervention in a population at-risk for household food insecurity.

## 6. Conclusions

We found that the Food FARMacia reached its target population, and a clinically based mobile food pantry program and enrollment in a concomitant research study was feasible. As healthcare systems increasingly focus on addressing upstream determinants of health to improve population health, scalable and effective interventions to reduce household food security should leverage the frequent routine well-child visits in the first years of life. Our findings support the need to conduct future, rigorous research to examine the effectiveness of clinically based mobile food pantry programs to reduce household food insecurity, improve diet quality, and improve health outcomes among families with household food insecurity in a primary care pediatrics setting. 

## Figures and Tables

**Table 1:** Baseline characteristics of families with child aged <6 years with household food insecurity screening at ambulatory care visit (2 April–2 August 2019).

Child Characteristics	Overall Patient Population (N = 650)	Patients with Household Food Insecurity (N = 172)	Food FARMacia Participants (N = 50)
Child Age, mean (SD), months	19.6 (20.5)	21.1 (22.1)	22.0 (18.4)
Child Age, n (%), years			
0 to <1	324 (49.8)	84 (49)	17 (34)
1 to <2	107 (16.5)	25 (15)	12 (24)
2 to <6	219 (33.7)	63 (37)	21 (42)
Female, n (%)	326 (50.2)	88 (51)	20 (40)
Race/ethnicity, n (%) ^a^			
Hispanic/Latino	546 (90.5)	148 (91)	44 (88)
White, not Hispanic/Latino	15 (2.5)	1 (1)	1 (2)
Black, not Hispanic/Latino	28 (4.7)	10 (6)	4 (8)
Other, not Hispanic/Latino	14 (2.3)	4 (2)	1 (2)
Household Characteristics			
Household size, mean (SD)	4.1 (1.4)	4.2 (1.5)	4.2 (1.2)
Annual household income, n (%)			
<USD 10 k	174 (26.8)	58 (34)	18 (36)
USD 10 k to <20 k	166 (25.5)	44 (26)	10 (20)
>USD 20 k	225 (34.6)	38 (22)	9 (18)
Missing	85 (13.1)	32 (18)	13 (26)
**Health Related Social Needs (HRSN)**			
≥2 ED visits past year, n (%) ^d^	120 (18.7)	40 (24)	11 (25)
HRSN Type, n (%)			
Household food insecurity	172 (26.5)	172 (100)	50 (100)
Housing needs ^b^	133 (20.7)	74 (45)	18 (39)
Kitchen problems ^b^	51 (7.9)	22 (13)	1 (2)
Transportation needs ^c^	68 (10.6)	47 (29)	7 (16)
Utility needs ^d^	40 (6.2)	27 (16)	7 (16)
Safety concerns ^b^	7 (1.1)	5 (3)	1 (2)
Number of HRSN, n (%)			
None	394 (60.6)	0	0
1 HRSN	145 (22.3)	71 (41)	25 (50)
2 HRSN	71 (10.9)	62 (36)	19 (38)
3 HRSN	30 (4.6)	29 (17)	4 (8)
4 HRSN	7 (1.1)	7 (4)	2 (4)
5 HRSN	3 (0.5)	3 (2)	0

^a^ N = 603 overall, N = 163 for household food insecurity, and N = 50 for Food FARMacia; ^b^ N = 644 overall, N = 166 for household food insecurity, and N = 46 for Food FARMacia; ^c^ N = 641 overall, N = 163 for household food insecurity, and N = 43 for Food FARMacia; ^d^ N = 642 overall, N = 164 for household food insecurity, and N = 44 for Food FARMacia.

**Table 2:** Baseline characteristics of longitudinal survey study participants according to retention in Food FARMacia intervention at 6 months.

		Food FARMacia at 6 Months		
	Overall (N = 48)	Retained (N = 32)	Attrition (N = 16)	*p*-Value ^a^
Child Baseline Characteristics				
Child Age, mean (SD), months	21.8 (18.4)	26.7 (18.7)	12.1 (13.8)	0.01
Child Age, n (%), years				0.01
0 to <1	17 (35)	9 (28)	8 (50)	
1 to <2	11 (23)	5 (16)	6 (38)	
2 to <6	20 (42)	18 (56)	2 (12)	
Female, n (%)	19 (40)	12 (38)	7 (44)	0.92
Race/ethnicity, n (%)				<0.01
Hispanic/Latino	42 (88)	31 (97)	11 (69)	
White, not Hispanic/Latino	1 (2)	1 (3)	0 (0)	
Black, not Hispanic/Latino	4 (8)	0 (0)	4 (25)	
Other, not Hispanic/Latino	1 (2)	0 (0)	1 (6)	
**Parental Baseline Characteristics**				
Female, n (%)	48 (100)	32 (100)	16 (100)	1.00
Spanish Language, n (%)	36 (75)	26 (81)	10 (63)	0.18
Education level				0.54
Elementary School or less	11 (23)	9 (28)	2 (12)	
Some High School	11 (23)	6 (19)	5 (31)	
High School Graduate	15 (31)	11(34)	4 (25)	
Some college	7 (15)	4 (12)	3 (19)	
College graduate	4 (8)	2 (6)	2 (12)	
**Household Characteristic**				
Household size, mean (SD)	4.2 (1.3)	4.5 (1.1)	3.7 (1.4)	0.04
Annual household income, n (%)				0.29
<USD 10 k	18 (38)	10 (31)	8 (50)	
USD 10 k or more	17 (35)	13 (41)	4 (25)	
Missing/Unknown	13 (27)	9 (28)	4 (25)	
WIC Enrollment, Yes, n (%)	47 (98)	31 (97)	16 (100)	1.00
SNAP Enrollment, n (%)				0.16
Yes	26 (54)	17 (53)	9 (56)	
No, but received in past	12 (25)	6 (19)	6 (38)	
Never	10 (21)	9 (28)	1 (6)	

^a^*p*-value from univariate tests (Wilcoxon rank-sum for continuous variables and Fisher’s exact test for categorical variables as appropriate).

## Data Availability

The data presented in this study are available upon request from the corresponding author. The data are not publicly available due to privacy restrictions.

## References

[1] Office of Disease Prevention and Health Promotion Social Determinants of Health. https://www.healthypeople.gov/2020/topics-objectives/topic/social-determinants-of-health.

[2] IOM (Institute of Medicine) (2014). Capturing Social and Behavioral Domains and Measures in Electronic Health Records: Phase 2.

[3] Daniel H., Bornstein S.S., Kane G.C. (2018). Addressing Social Determinants to Improve Patient Care and Promote Health Equity: An American College of Physicians Position Paper. Ann. Intern. Med..

[4] Council on Community Pediatrics (2016). Poverty and Child Health in the United States. Pediatrics.

[5] De Marchis E.H., Torres J.M., Benesch T., Fichtenberg C., Allen I.E., Whitaker E.M., Gottlieb L.M. (2019). Interventions Addressing Food Insecurity in Health Care Settings: A Systematic Review. Ann. Fam. Med..

[6] Crossa A., Baquero M., Etheredge A.J., Seidl L., Nieves C., Dannefer R., Solomon E., Prasad D., Jasek J., Dongchung T.Y. (2021). Food Insecurity and Access in New York City during the COVID-19 Pandemic, 2020. https://www1.nyc.gov/assets/doh/downloads/pdf/epi/databrief128.pdf.

[7] Suglia S.F., Duarte C.S., Chambers E.C., Boynton-Jarrett R. (2012). Cumulative social risk and obesity in early childhood. Pediatrics.

[8] Hemmingsson E., Johansson K., Reynisdottir S. (2014). Effects of childhood abuse on adult obesity: A systematic review and meta-analysis. Obes. Rev..

[9] Gluckman P.D., Hanson M.A., Bateson P., Beedle A.S., Law C.M., Bhutta Z.A., Anokhin K.V., Bougneres P., Chandak G.R., Dasgupta P. (2009). Towards a new developmental synthesis: Adaptive developmental plasticity and human disease. Lancet.

[10] Gluckman P.D., Hanson M.A., Cooper C., Thornburg K.L. (2008). Effect of in utero and early-life conditions on adult health and disease. N. Engl. J. Med..

[11] Barker D.J., Osmond C. (1986). Infant mortality, childhood nutrition, and ischaemic heart disease in England and Wales. Lancet.

[12] Food Research & Action Center https://frac.org/aaptoolkit.

[13] Black M.M., Quigg A.M., Cook J., Casey P.H., Cutts D.B., Chilton M., Meyers A., Ettinger de Cuba S., Heeren T., Coleman S. (2012). WIC participation and attenuation of stress-related child health risks of household food insecurity and caregiver depressive symptoms. Arch. Pediatrics Adolesc. Med..

[14] Gany F., Lee T., Loeb R., Ramirez J., Moran A., Crist M., McNish T., Leng J.C. (2015). Use of Hospital-Based Food Pantries among Low-Income Urban Cancer Patients. J. Community Health.

[15] Berkowitz S.A., Delahanty L.M., Terranova J., Steiner B., Ruazol M.P., Singh R., Shahid N.N., Wexler D.J. (2019). Medically Tailored Meal Delivery for Diabetes Patients with Food Insecurity: A Randomized Cross-over Trial. J. Gen. Intern. Med..

[16] Bryce R., Guajardo C., Ilarraza D., Milgrom N., Pike D., Savoie K., Valbuena F., Miller-Matero L.R. (2017). Participation in a farmers’ market fruit and vegetable prescription program at a federally qualified health center improves hemoglobin A1C in low income uncontrolled diabetics. Prev. Med. Rep..

[17] Freedman D.A., Choi S.K., Hurley T., Anadu E., Hébert J.R. (2013). A farmers’ market at a federally qualified health center improves fruit and vegetable intake among low-income diabetics. Prev. Med..

[18] Downer S., Berkowitz S.A., Harlan T.S., Olstad D.L., Mozaffarian D. (2020). Food is medicine: Actions to integrate food and nutrition into healthcare. Bmj.

[19] Wright B.N., Bailey R.L., Craig B.A., Mattes R.D., McCormack L., Stluka S., Franzen-Castle L., Henne B., Mehrle D., Remley D. (2018). Daily Dietary Intake Patterns Improve after Visiting a Food Pantry among Food-Insecure Rural Midwestern Adults. Nutrients.

[20] Martin K.S., Wu R., Wolff M., Colantonio A.G., Grady J. (2013). A novel food pantry program: Food security, self-sufficiency, and diet-quality outcomes. Am. J. Prev. Med..

[21] Eicher-Miller H.A. (2020). A review of the food security, diet and health outcomes of food pantry clients and the potential for their improvement through food pantry interventions in the United States. Physiol. Behav..

[22] An R., Wang J., Liu J., Shen J., Loehmer E., McCaffrey J. (2019). A systematic review of food pantry-based interventions in the USA. Public Health Nutr..

[23] Marmash D., Ha K., Sakaki J.R., Gorski I., Rule B., Foster J., Puglisi M., Chun O.K. (2021). Diet Quality, Nutritional Adequacy, and Sociodemographic Characteristics of Mobile Food Pantry Users in Northeastern Connecticut. Nutrients.

[24] City of New York New York City Census Fact Finder. https://popfactfinder.planning.nyc.gov/#12.25/40.724/-73.9868.

[25] Billioux A., Verlander K., Anthony S., Alley D.E. (2017). Standardized Screening for Health-Related Social Needs in Clinical Settings: The Accountable Health Communities Screening Tool.

[26] Hager E.R., Quigg A.M., Black M.M., Coleman S.M., Heeren T., Rose-Jacobs R., Cook J.T., Ettinger de Cuba S.A., Casey P.H., Chilton M. (2010). Development and validity of a 2-item screen to identify families at risk for food insecurity. Pediatrics.

[27] Smith S., Malinak D., Chang J., Perez M., Perez S., Settlecowski E., Rodriggs T., Hsu M., Abrew A., Aedo S. (2017). Implementation of a food insecurity screening and referral program in student-run free clinics in San Diego, California. Prev. Med. Rep..

[28] Hickey E., Phan M., Beck A.F., Burkhardt M.C., Klein M.D. (2020). A Mixed-Methods Evaluation of a Novel Food Pantry in a Pediatric Primary Care Center. Clin. Pediatrics.

[29] Center on Budget and Policy Priorities. https://www.cbpp.org/research/food-assistance/wic-works-addressing-the-nutrition-and-health-needs-of-low-income-families.

[30] Masciale M., Lopez M.A., Yu X., Domínguez J., Fredricks K., Haq H., Raphael J.L., Bocchini C. (2021). Public Benefit Use and Social Needs in Hospitalized Children with Undocumented Parents. Pediatrics.

[31] Pelto D.J., Ocampo A., Garduño-Ortega O., Barraza López C.T., Macaluso F., Ramirez J., González J., Gany F. (2020). The Nu-trition Benefits Participation Gap: Barriers to Uptake of SNAP and WIC among Latinx American Immigrant Families. J. Community Health.

[32] Leung C.W., Hoffnagle E.E., Lindsay A.C., Lofink H.E., Hoffman V.A., Turrell S., Willett W.C., Blumenthal S.J. (2013). A qual-itative study of diverse experts’ views about barriers and strategies to improve the diets and health of Supplemental Nutrition Assistance Program (SNAP) beneficiaries. J. Acad. Nutr. Diet..

[33] Fan L., Gundersen C., Baylis K., Saksena M. (2021). The Use of Charitable Food Assistance among Low-Income Households in the United States. J. Acad. Nutr. Diet..

[34] Twersky S.E. (2019). Restrictive state laws aimed at immigrants: Effects on enrollment in the food stamp program by U.S. citizen children in immigrant families. PLoS ONE.

[35] Ettinger de Cuba S.A., Bovell-Ammon A.R., Cook J.T., Coleman S.M., Black M.M., Chilton M.M., Casey P.H., Cutts D.B., Heeren T.C., Sandel M.T. (2019). SNAP, Young Children’s Health, and Family Food Security and Healthcare Access. Am. J. Prev. Med..

[36] Criss S., Woo Baidal J.A., Goldman R.E., Perkins M., Cunningham C., Taveras E.M. (2015). The Role of Health Information Sources in Decision-Making among Hispanic Mothers during Their Children’s First 1000 Days of Life. Matern. Child Health J..

